# Supplementary material for: Influence of shape resonances on the angular dependence of molecular photoionization delays
Source: Nat Commun. 2021 Dec 20;12:7343. doi: 10.1038/s41467-021-27360-y (PMC8688504; doi:10.1038/s41467-021-27360-y)
Supplement: Supplementary file 1 — Supplementary Information [file 41467_2021_27360_MOESM1_ESM.pdf]

Supplementary Information

Influence of Shape Resonances on the Angular Dependence of Molecular Photoionization Delays

F. Holzmeier et al.

Table of Contents

Supplementary Note 1: Experimental setup 2

Supplementary Note 2: Extraction of experimental MFPADs 2

Supplementary Note 3: Photoionization dipole amplitudes and time delays in the molecular frame 3

Supplementary Note 4: Extraction of experimental DMEs from measured MFPADs 4

Supplementary Note 5: Effect of using relative phases 6

Supplementary Note 6: Calculation of the partial-wave dipole matrix elements and scattering *S*-matrix. 7

Supplementary Note 7: One-dimensional representation of the PDA magnitude, phase and delay 9

Supplementary Note 8: Comportments of the PDA curves in the complex plane in the Fano model .. 10

Supplementary References 11

Supplementary Note 1: Experimental setup

The monochromatized synchrotron radiation has a bandwidth of around 2.5 meV in the employed energy range and is focused at the center of a COLTRIMS-type 3D electron-ion coincidence momentum spectrometer. This set-up, derived from earlier versions[1,2], is optimized here to investigate dissociative photoionization of molecules which occurs at the intersection of the light beam and a supersonic molecular beam generated through a 70 µm nozzle and two skimmers (500 and 700 µm diameter). Ions and electrons, guided towards time and position sensitive delay line detectors (RoentDek DLD-80) by a set of variable electrostatic and magnetic fields, combined with an electrostatic lens focusing the trajectories of energetic ion fragments[3], are collected over a 4π solid angle. In the study of dissociative photoionization of NO, the velocity vectors $\mathbf{v}_{N+}$ and $\mathbf{v}_e$ of N^+ ions and photoelectrons are derived from the impact positions and time of flight at the detectors employing a multichannel time to digital converter for positions and ion time of flight and a synchronized time to amplitude converter for the electron time of flight[3].

Supplementary Note 2: Extraction of experimental MFPADs

In this work, we investigate photoionization of $NO(X^2\Pi)$ to the $NO^+(c^3\Pi, \mathrm{v}=0)$ state, with the ionization potential IP=21.73 eV corresponding to ionization from the 4σ inner valence molecular orbital, dissociating to the N^+ (^3P) + O(^3P) limit at 21.03 eV relative to the $NO(X^2\Pi, \mathrm{v}=0)$ origin. In brief, the coincident events (N^+,e) corresponding to this DPI process were selected from the electron-ion kinetic-energy correlation histogram, and the MFPAD $I(\hat{k}, \hat{\Omega}) = I(\theta_k, \phi_k, \chi, \gamma)$ was constructed from the ($\mathbf{v}_{N+}$, $\mathbf{v}_e$) ion and photoelectron velocity vectors, as described in [4] and references therein, within the axial-recoil approximation[5]. $\hat{k} \equiv (\theta_k, \phi_k)$ defines the emission direction in the molecular frame with θ_k and ϕ_k being the polar and azimuthal angles of $\mathbf{v}_e$ relative to $\mathbf{v}_{N+}$. The Euler angles $\hat{\Omega} \equiv (\gamma, \chi, \beta)$ with the polar and azimuthal angle of $\mathbf{v}_{N+}$ relative to the light quantization axis $\hat{\mathbf{e}}$ χ and γ, define the orientation of the molecular axis, i.e., the rotation of the molecular frame into the field frame (FF).

The MFPADs are expanded using the F_{LN}-function formalism developed previously[6,7]. For dissociative photoionization of linear molecules induced by circularly polarized light, within the dipole approximation, the $I(\theta_k, \phi_k, \chi)$ MFPAD is determined by five one-dimensional $F_{LN}(\theta_k)$ functions according to the general expression[7]:

$$\begin{aligned} I_{\pm}(\theta_k, \phi_k, \chi) = F_{00}(\theta_k) &- \frac{1}{2} F_{20}(\theta_k) P_2^0(\cos\chi) \\ &- \frac{1}{2} F_{21}(\theta_k) P_2^1(\cos\chi)\cos\phi_k \\ &- \frac{1}{2} F_{22}(\theta_k) P_2^2(\cos\chi)\cos(2\phi_k) \\ &\pm F_{11}(\theta_k) P_1^1(\cos\chi)\sin\,\phi_k \end{aligned} \tag{S1}$$

where P_L^N are the associated Legendre polynomials. The F_{LN} functions and their statistical uncertainties extracted from the fit of the measured $I(\theta_k, \phi_k, \chi)$ distribution according to Eq. (S1) enable us to reconstruct the MF emission diagram $I_\chi(\theta_k, \phi_k)$ for any orientation of the molecular axis relative to the quantization axis of circularly or linearly polarized light. In particular, for a molecular orientation parallel to the polarization of linearly polarized light considered to characterize the role of the $4\sigma \rightarrow k\sigma^*$ shape resonance, the MFPAD is fully described with

$$I_{\chi=0^\circ}(\theta_k) = F_{00}(\theta_k) + F_{20}(\theta_k). \tag{S2}$$

and, e.g., for a molecular perpendicular orientation it writes as

$$I_{\chi=90^\circ}(\theta_k,\phi_k) = F_{00}(\theta_k) - 0.5F_{20}(\theta_k) + 3F_{22}(\theta_k)\cos(2\phi_k). \quad \text{(S3)}$$

Experimental data were recorded at eleven photon energies corresponding to the harmonics of a 800 nm Ti:Sa laser typically used in attosecond XUV+IR RABBITT-type experiments between 23.25 eV (H15) and 38.75 eV (H25), plus the 22.5 eV and 48.4 eV energies.

Supplementary Note 3: Photoionization dipole amplitudes and time delays in the molecular frame

The MFPAD $I(\hat{k},\hat{\Omega},E)$, i.e., the angular photoemission probability in the molecular frame (MF) for fixed-in-space molecules, is proportional to the absolute square of the photoionization amplitude $D(\hat{k},\hat{\Omega},E)$,

$$I(\hat{k},\hat{\Omega},E) = \frac{4\pi^2 h\nu}{c}\left|D(\hat{k},\hat{\Omega},E)\right|^2. \quad \text{(S4)}$$

The photoionization amplitude can be written as

$$D(\hat{k},\hat{\Omega},E) = \left\langle \Psi_{\hat{k}}^{(-)}(E)\middle| \vec{r}\cdot\vec{E}\,(\hat{\Omega})\middle|\Psi_0\right\rangle \quad \text{(S5)}$$

where $\Psi_{\hat{k}}^{(-)}$ is the wave function of the final state for the emitted photoelectron in the MF with a kinetic energy E, Ψ_0 is the initial state, $\vec{r}$ the dipole operator, $\vec{E}$ is the electric field. In the dipole approximation, with circularly polarized light propagating in the $\hat{z}_{\mathrm{FF}}$ direction and helicity $h=\pm1$, or with light linearly polarized in the $\hat{x}_{\mathrm{FF}} \equiv (\chi,\gamma)$ direction, for which we take $h=0$, the corresponding PDA, can be written as

$$D(\hat{k},\hat{\Omega},E) = \sum_{\mu}\left\langle \Psi_{\hat{k}}^{(-)}(E)\middle|r_\mu\middle|\Psi_0\right\rangle R_{\mu,h}^{(1)}(\hat{\Omega}). \quad \text{(S6)}$$

where $R_{\mu,h}^{(1)}(\hat{\Omega})$ is a rotation matrix and $r_\mu = \sqrt{\frac{4\pi}{3}}\,rY_{1,\mu}(\theta,\phi)$ is the dipole operator with μ being the projected angular momentum of the photon on the molecular axis. The N electron final scattering state including the $(N-1)$ ion state and the photoelectron is currently expanded in partial waves as

$$\Psi_{\hat{k}}^{(-)}(E) = e^{-i\sigma_0}\sum_{l,m} i^l e^{-i\Delta\sigma_l}\,\psi_{l,m}^{(-)}(E)Y_{l,m}^{*}(\hat{k}) \quad \text{(S7)}$$

where the σ_l are the Coulomb scattering phases, $\sigma_l = \arg\,\Gamma\left(l+1-\frac{i}{k}\right)$, and $\Delta\sigma_l = \sigma_l - \sigma_0$. Note that σ_0 is singular as $k\to 0$ and behaves as

$$\sigma_0 \to \frac{1}{k}(\ln k + 1) - \frac{\pi}{4} \quad \text{(S8)}$$

where k is the photoelectron momentum $E = \left(\frac{1}{2}\right)k^2$, but the $\Delta\sigma_l$ are all finite at k=0. The dipole matrix element for each partial wave (DMEs) is defined as

$$\begin{aligned} D_{l,m,\mu}(E) &= e^{i\sigma_0}(-i)^l e^{i\Delta\sigma_l}\left\langle \psi_{l,m}^{(-)}(E)\middle|r_\mu\middle|\Psi_0\right\rangle \\ &= d_{l,m,\mu}e^{i\eta_{l,m,\mu}} \end{aligned} \quad \text{(S9)}$$

where $d_{l,m,\mu}$ is the real-valued magnitude of the DME and $\eta_{l,m,\mu}$ is its phase.
Combining Eq. (S6-S9) leads to the expression of the PDA as given in Eq. (2) of the main paper:

$$D(\hat{k},\hat{\Omega},E) = \sum_{\mu}\sum_{l,m} D_{l,m,\mu}(E) Y_{l,m}(\hat{k}) R^{(1)}_{\mu,h}(\hat{\Omega}) \tag{S10}$$

This provides then the one-photon ionization time delay $\tau(\hat{k},\hat{\Omega},E)$ as the energy derivative of its argument $\eta(\hat{k},\hat{\Omega},E)$:

$$\tau(\hat{k},\hat{\Omega},E) = \frac{\mathrm{d}\arg\left(D(\hat{k},\hat{\Omega},E)\right)}{\mathrm{d}E} = \frac{\mathrm{d}\eta(\hat{k},\hat{\Omega},E)}{\mathrm{d}E} \tag{S11}$$

Supplementary Note 4: Extraction of experimental DMEs from measured MFPADs

The determination of the partial-wave dipole matrix elements $D_{l,m,\mu}$ relies on the expansion of the five F_{LN} functions in Legendre polynomials:

$$F_{LN}(\theta_k) = \sum_{L'=0}^{2l_x} C_{L'LN} P_{L'}^{N}(\cos\theta_k), \tag{S12}$$

where the $C_{L'LN}$ coefficients are expressed in terms of the DMEs as

$$C_{L'LN} = \frac{2\pi h\nu}{c} \sum_{\substack{l,m,\mu \\ l',m',\mu'}} (-1)^{m'+\mu'} \left[\frac{(2l+1)(2l'+1)(L'-N)!\,(L-N)!}{(L'+N)!\,(L+N)!}\right]^{\frac{1}{2}} \times D_{l,m,\mu} D^{*}_{l',m',\mu'} \langle ll'00|L'0\rangle\langle l,l',m,-m'|L'N\rangle \times \langle 1,1,\mu,-\mu'|L,N\rangle f_{N,L}$$

$$f_{N,L} = \begin{cases} \frac{1}{2}\langle 1,1,0,0|L,0\rangle & \text{if } N=0 \\ \langle 1,1,0,0|L,0\rangle & \text{if } L=2 \text{ and } N\neq 0 \\ i\langle 1,1,-1,1|L,0\rangle & \text{if } L=1 \text{ and } N=1 \end{cases} \tag{S13}$$

Note that in the studied reaction, to a very good approximation, only $m=\mu$ DMEs contribute to the PDA, so we drop the μ label in the following.
For each photon energy, a non-linear least-squares fit of the experimental data, as described in[6,7], is performed giving access to the magnitudes $d_{l,m}$ and phases $\eta_{l,m}$ of the one-photon ionization dipole matrix elements. The experimental $F_{LN}(\theta_k)$ functions (Eq. (S12)) were used to generate a set of MFPAD data for a range of θ_k angles, with the light quantization axis being parallel, perpendicular, and at the magic angle with respect to the molecular axis, and with both linear and circular polarization, for a total of 113 data points that were fit at each photon energy. The parameter space of possible dipole matrix elements was sampled, usually with 10,000 random initial parameter sets, with the only constraint being that the total cross section was the same as that of the data being fit. The parameters at the nearest local minimum of the sum of the squares of the deviations, starting from each initial parameter set, were then found using the Levenberg–Marquardt algorithm. For the studied energies, a single local minimum, or two local minima corresponding to a similar quality of the fits, were found, which were significantly better than all the other fits[7]. In the presence of two solutions, the selection was based on a criterion of closest resemblance with the calculation. The statistical uncertainties of the DMEs were deduced from the root-mean-square (rms) deviation of the fit.

The MFPADs measured at different excitation energies were normalized relative to the total cross section for PI into the $NO^+(c^3\Pi)$ ionic state[8,9]: here we chose to calibrate the measured C_{000} Legendre polynomial coefficients to those obtained in the reported calculations. On the other hand, we have used the phase of a selected partial-wave DME, here the $\eta_{2,1}$ phase of the $(d\pi)$ partial wave, which is not affected by the shape resonance (sec. III.), as a phase reference for all DMEs at the different excitation energies, such that $\tilde{\eta}_{lm} = \eta_{lm} - \eta_{21}$. Choosing such a common reference establishes the desired consistency between the different energies. It also removes the low energy divergence of the Coulomb phase from the phase of the PDAs. The effect of using such relative phases is discussed further in Supplementary Note 5. We note that, if desired, the part of the Coulomb phase that was subtracted with the reference phase could be reintroduced by multiplying all DMEs by $\exp(i\sigma_{l=2})$ (see Eq. (S9)).

Fig. S1 shows the experimental and computed DME magnitudes and phases as a function of the electron/photon energy. Relying on the smooth dependence of the DMEs across a broad shape resonance (see also Fig. S1 b,d for the theory), a spline fit of the experimental data was used to obtain the PDAs on a finer energy grid. To improve the fit at the boundaries of the eleven main data points, additional data points recorded at 22.5 eV and 48.4 eV were used.

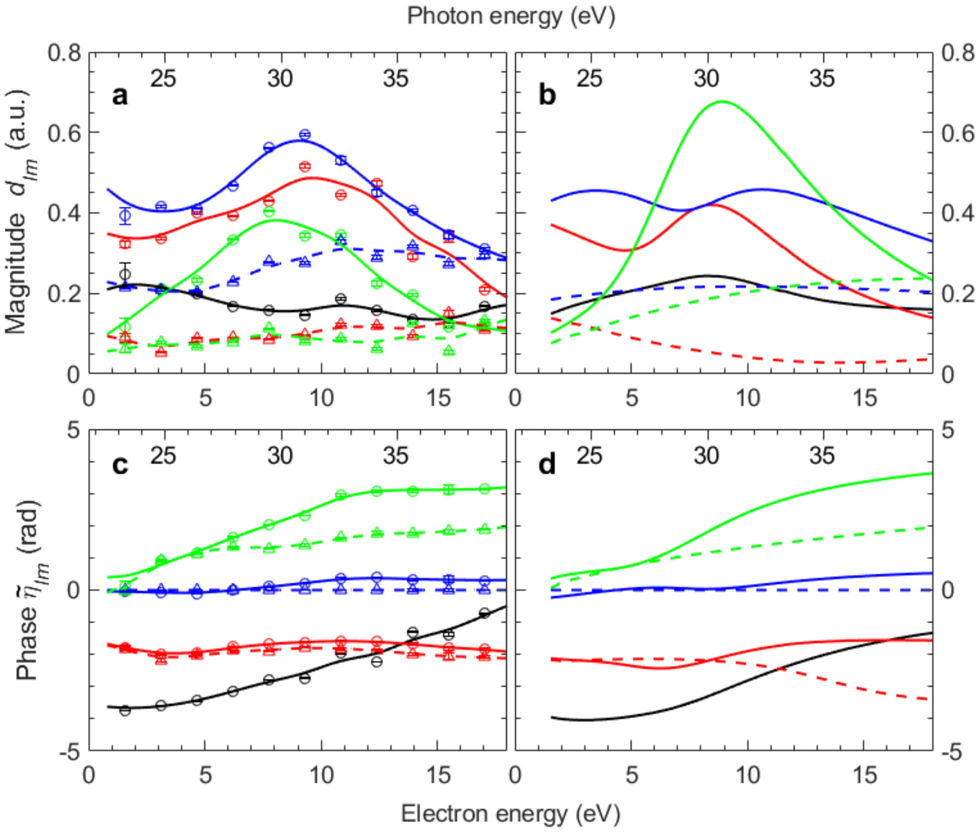

Fig. S1 Magnitudes and phases of the partial wave dipole matrix elements. **a**,**c** Experimental and **b**,**d** computed magnitudes and phases of the partial-wave DMEs as a function of the photoelectron (and photon) energy for different values of (l, m), (black line (0,0); red line: (1,0); blue line: (2,0); green line: (3,0); red dashed line: (1,1); blue dashed line: (2,1); green dashed line: (3,1)). Error bars (two standard deviations) feature the statistical uncertainties in extracting the DME magnitudes and phases. The full and dashed lines in **a** and **c** are spline fits to the experimental data. The phases $\tilde{\eta}_{lm}$ are relative to the phase η_{21}.

The magnitudes of the $(l,0) \equiv (l\sigma)$ DMEs, describing the parallel transition, dominate the explored energies, with a characteristic strong contribution of the $(f\sigma)$ partial wave from the $4\sigma \rightarrow k\sigma^*$ shape resonance clearly identified both in the measured and computed DMEs. The $(p\sigma)$ and $(d\sigma)$ partial waves are also found to take part in the resonance for the measured MFPADs. All extracted phases are referenced to the phase of the $(d\pi)$ partial wave, which is not affected by the resonance and displays a slow variation with the energy (see Fig. S2): $\tilde{\eta}_{lm} = \eta_{lm} - \eta_{21}$. The $\tilde{\eta}_{lm}$ phases do not include the low energy singularity which comes from the long-range Coulomb potential.

Supplementary Note 5: Effect of using relative phases

In Fig. S2, we give the computed absolute DME phases including the Coulomb phases for the photoionization of NO to the $c\ ^3\Pi$ state of NO^+. The effect of the Coulomb phase is most noticeable at low energies. The non-Coulomb phase of the (*l*,*m*)=(2,1) DME, whose magnitude dominates the perpendicular transition unaffected by the σ^* shape resonance (cf. Fig. S1), displays a weak variation in the studied energy range. It is chosen as a reference for the phases of all (*l*,*m*) DMEs providing the $\tilde{\eta}_{lm} = \eta_{lm} - \eta_{21}$ phases reported in Fig. S1c and d. This subtraction removes the divergence in the Coulomb phase at low energy from the phase of each partial-wave DME.

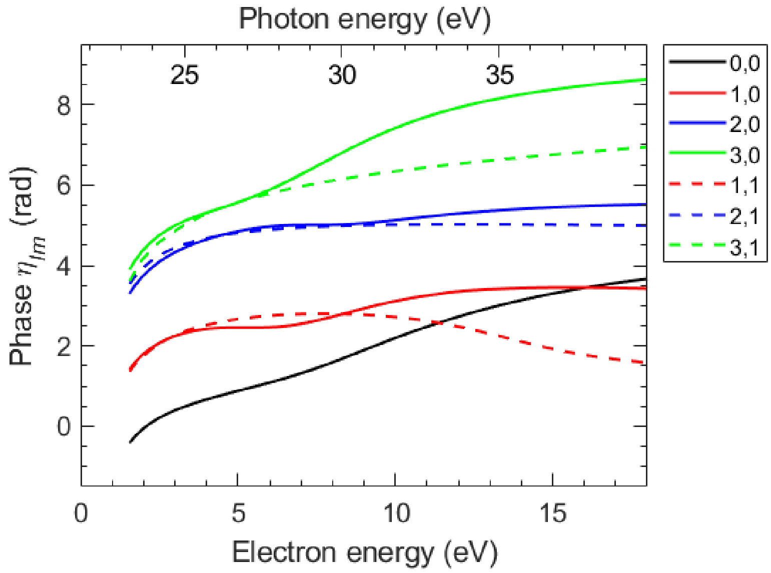

Fig. S2 Computed absolute DMEs. Variation of the computed absolute phases of the partial-wave dipole matrix elements, defined in Eq. (2), as a function of electron energy.

Fig. S3 shows a comparison of the computed absolute phases of the PDAs (a) and the corresponding time delays (c) obtained as their energy derivatives, with those obtained by summing the partial-waves DMEs referenced to the $\eta_{2,1}$ phase (b,d). One sees that in terms of photoionization time delays, adding back in the Coulomb phase results in adding a large and positive time delay (several hundreds of as) at low photoelectron kinetic energy, independent of the emission direction.

Taking such a reference thus removes the rapid rise of the Coulomb phase at low electron energies and the resulting positive contribution to the time delays, highlighting specifically the influence of the scattering from the short-range molecular potential and the dipole coupling from which the electron is removed. As stated in Note 4, the standard contribution of the Coulomb phase can be added back in analytically if desired (cf. Eq. (S9)).

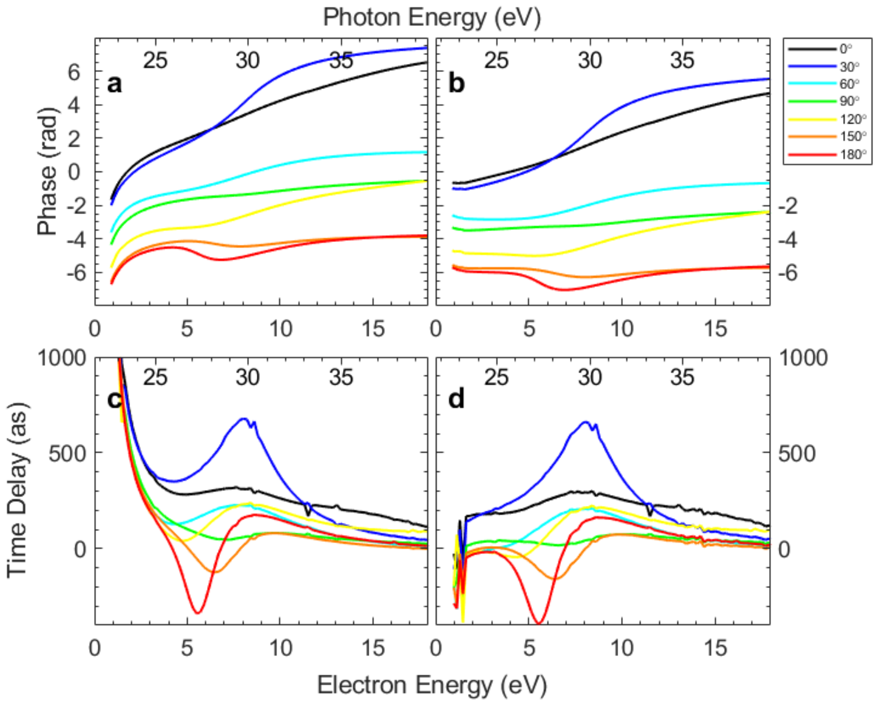

Fig. S3 Computed absolute PDA phases and ionization delays for fixed emission directions. The full absolute phases and time delays of the computed PDAs near threshold are presented for fixed emission directions in the molecular frame. **a**,**c** Absolute phases including Coulomb phases and corresponding ionization delays. **b**,**d** Computed results relative to $\eta_{2,1}$.

Supplementary Note 6: Calculation of the partial-wave dipole matrix elements and scattering *S*-matrix.

The DMEs reported in Fig. S1 providing the theoretical PDAs and photoionization time delays presented in Fig. 2 and Fig. S4 for photoionization of $\mathrm{NO}(X^2\Pi)$ to the $c\ ^3\Pi$ state of NO^+, corresponding to ionization from the 4σ inner valence molecular orbital, were computed using the multichannel Schwinger configuration interaction method as described in [4]. Briefly, the calculations utilized a single-center expansion, with $l_{\max} = 60,$ and the ion and initial states were given by complete active space configuration interaction wave functions with 8 active orbitals (four σ orbitals and four π orbitals) obtained from a valence complete active space self-consistent field calculation on the ground state of the molecule. They were performed at a fixed inter-nuclear distance of 1.15077 Å[10] (experimental r_e) with an augmented correlation-consistent polarized valence triple-zeta basis[11,12] to represent the bound orbitals. The photoionization calculation[8,13] used a close-coupling expansion of the photoionized state, including ten open channels with ion states of vertical ionization potentials near or below the ionization potential of the $c\ ^3\Pi$ state. The states included were the $X\ ^1\Sigma^+$, $a\ ^3\Sigma^+$, $b\ ^3\Pi$, $w\ ^3\Delta$, $b'\ ^3\Sigma^-$, $A'\ ^1\Sigma^-$, $A\ ^1\Pi$, $W\ ^1\Delta$, $c\ ^3\Pi$, and $B\ ^1\Pi$ states. These calculations successfully describe the role of electronic correlations in photoionization of NO leading to the $c\ ^3\Pi$ state, as well as the $4\sigma \rightarrow k\sigma^*$ shape resonance which affects the parallel component of the transition[7].

In order to extract the resonant scattering time delay coming only from the shape resonance in the $c\ ^3\Pi$ channel, we used a one-channel scattering calculation restricted to the $c\ ^3\Pi$ state of NO^+ and to the σ

symmetry. The boundary conditions in the scattering calculation were for the $\psi_k^{(+)}$ scattering state (i.e. normal scattering state) which has an incoming Coulomb wave with asymptotic momentum $\vec{k}$ and outgoing spherical scattered Coulomb waves. The number of partial waves was the same as in the photoionization calculations but the amplitudes were given by the scattering *S*-matrix and not by the photoionization transition amplitudes. Indeed, when using the same ten-state wave function expansion for the *e*-ion scattering calculation as was used in the photoionization calculation, there were contributions from a number of overlapping broad resonances coming from scattering from the different ion states included. This led to an eigenphase sum from which it was difficult to extract the time delay just from the resonance from scattering from *c* $^3\Pi$ state of NO^{+}. The eigenphase sums from the single channel calculations as a function of scattering energy and half of the corresponding Wigner time delay are presented in Fig. 5.

Supplementary Note 7: One-dimensional representation of the PDA magnitude, phase and delay

Fig. S4 is a complementary representation of the 2D polar plot in Fig. 2 showing the magnitude, phase, and time delay along different emission directions for experiment and theory, respectively.

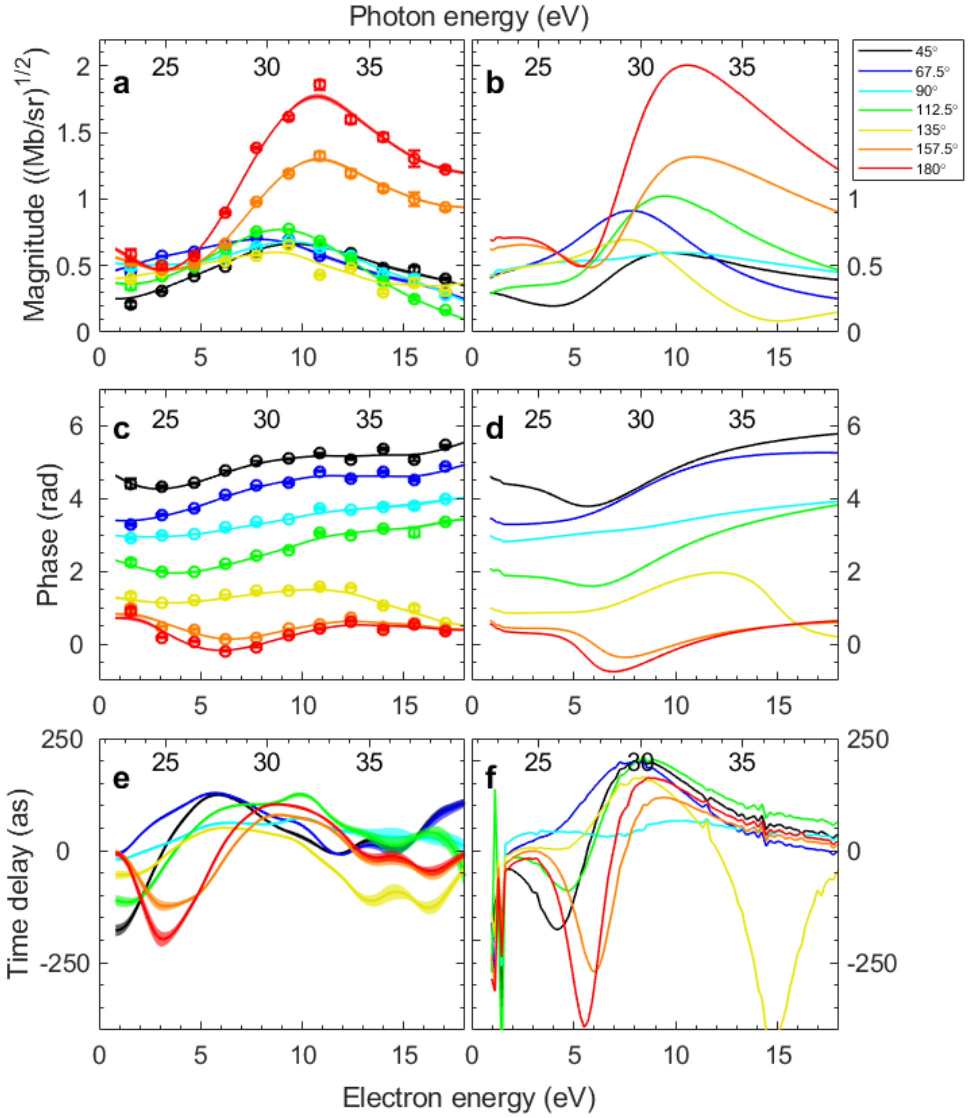

Fig. S4 Experimental and computed PDAs for selected angles. **a**, **c**, **e** Experimental and **b**, **d**, **f** computed PDAs and photoionization time delays as a function of electron (or photon) energy for selected emission angles and a molecular orientation parallel to the axis on linearly polarized light. Panels **a** and **b** represent the PDA magnitudes, panels **c** and **d** the PDA phases using the $\eta_{2,1}$ phase as a reference for the D_{lm} DME phases, and panels **e** and **f** the corresponding photoionization time delays. In (**a**, **c**, **e**) the thickness of the lines features the statistical uncertainties of the PDA magnitudes and phases, and that of the photoionization time-delays, deduced from those characterizing the magnitudes and phases of the DMEs in Fig. S1.

Supplementary Note 8: Comportments of the PDA curves in the complex plane in the Fano model

To illustrate some typical behaviors of the PDA and resulting time delays in the multichannel Fano model, we combine the expressions of the PDA summed over partial waves in Eq. (3) and that of each DME given in Eq. (5) with the additional assumption that the non-resonant DMEs, $D_l^{(0)}$, do not depend on energy. Then the behavior of the PDA is given by

$$D(\theta_k, \varepsilon) = D^{(0)}(\theta_k) + \alpha(\theta_k)\frac{q-i}{\varepsilon+i}. \tag{S14}$$

With these assumptions, the curves formed by the PDAs for fixed emission angles when the energy varies across the resonance are circles centered at $D_c = D^{(0)}(\theta_k) - \left(\frac{1}{2}\right)\alpha(\theta_k)(1+iq)$ with a radius of $R_c = \left(\frac{1}{2}\right)|\alpha(\theta_k)|\sqrt{1+q^2}$. The sources of the phase change in the PDAs can be made clear by rewriting Eq. (S14) as

$$D(\theta_k, \varepsilon) = D^{(0)}(\theta_k) - \alpha(\theta_k) + \alpha(\theta_k)(-q\sin\Delta + \cos\Delta)e^{i\Delta} \tag{S15}$$

where $\Delta = -\tan^{-1}\left(\frac{1}{\varepsilon}\right)$ is the scattering phase shift due to the short-range, non-Coulomb part of the electron-molecule interaction potential in the pure Breit-Wigner form for resonant scattering. Eq. (S15) shows that the phase change in the PDA comes from both the exponential term, $e^{i\Delta}$, and the term $(-q\sin\Delta + \cos\Delta)$ which is due to the interference between the resonant and non-resonant parts of the PDA. The exponential term by itself forms a curve that is a half-circle as ε goes from $-\infty \rightarrow +\infty$ so that its phase increases by π. Multiplying the exponential term by the interference term closes this curve to become a circle. The PDA then always starts and ends with the same phase if the infinite range of ε is considered, so that its phase will change by 0 or 2π depending on the radius of the circle and the location of its center. Examples for different parameters are displayed in Fig. S5. The circles formed by the PDAs in the complex plane are shown in Fig. S5a. The resulting time delays in panel (c) may be positive or negative, with the integral under the time delay profiles being 0 or 2π in agreement with the phase data presented in panel (b).

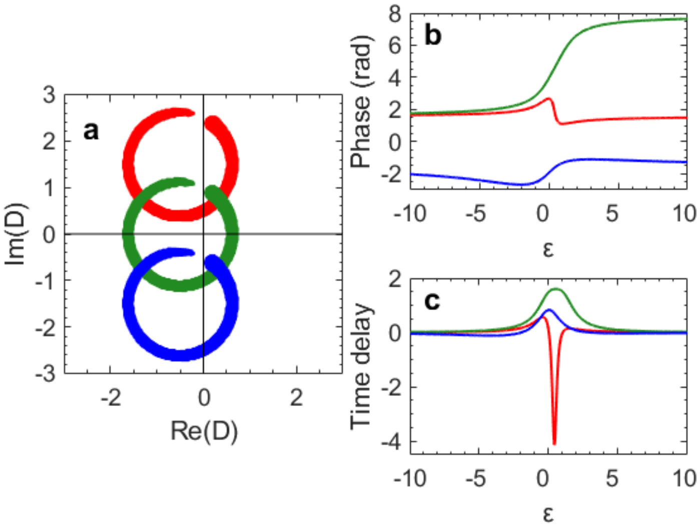

Fig. S5 Range of the behavior of PDA curves near a resonance. **a** Curves of the PDA for the model given in eq. (S14), for $q = 2.0, \alpha = 1.0$ and with the value of D^0 of $2.5i$ (red), $1.0i$ (green), and $-0.5i$ (blue), respectively. The increasing size of the markers indicates increasing value of the relative energy

ε. **b** The value of the phase of the PDA with the same set of D^0 values. **c** The energy derivative of the phases expressed as time delays. Note that in this example, since the energies are unitless quantities, the corresponding time delays are also unitless.

For the studied shape resonance in photoionization of NO, with $E_{\text{res}} \approx 8.5$ eV and $\Gamma \approx 7$ eV, the range of energies considered here is limited to about two times the width of the resonance. This leads to PDA phase changes ranging from 0 to 2π. The major photoemission process around ($\theta_k = 180°$) is a clear illustration of the model, with a time delay displaying both positive and negative values, as reported in Fig. 2c and Fig. S4e and f, and a net PDA phase change close to zero. The phase change in the PDA for 60° is closer to π. This behavior results from the nearly zero non-resonant PDA at 60°, as shown in Fig. 4b. The peak in the computed photoionization time delay at 60° (202 as), seen in Fig. 2c and Fig. S4f, is also very close to the time delay of the resonant PDA (237 as) reported in Fig. 4e.

Supplementary References

1. Gisselbrecht, M., Huetz, A., Lavollée, M., Reddish, T. J. & Seccombe, D. P. Optimization of Momentum Imaging Systems Using Electric and Magnetic Fields. *Rev. Sci. Instrum.* **76,** 13105 (2004).
2. Picard, Y. J. *et al.* Attosecond Evolution of Energy- and Angle-Resolved Photoemission Spectra in Two-Color (XUV + IR) Ionization of Rare Gases. *Phys. Rev. A* **89,** 31401 (2014).
3. Lebech, M., Houver, J. C. & Dowek, D. Ion–Electron Velocity Vector Correlations in Dissociative Photoionization of Simple Molecules Using Electrostatic Lenses. *Rev. Sci. Instrum.* **73,** 1866–1874 (2002).
4. Veyrinas, K. *et al.* Dissociative Photoionization of NO Across a Shape Resonance in the XUV Range Using Circularly Polarized Synchrotron Radiation. *J. Chem. Phys.* **151,** 174305 (2019).
5. Zare, R. N. Photoejection Dynamics. *Mol. Photochem.* **4,** 1–37 (1972).
6. Lucchese, R. R. *et al.* Polar and Azimuthal Dependence of the Molecular Frame Photoelectron Angular Distributions of Spatially Oriented Linear Molecules. *Phys. Rev. A* **65,** 20702 (2002).
7. Lebech, M. *et al.* Complete Description of Linear Molecule Photoionization Achieved by Vector Correlations Using the Light of a Single Circular Polarization. *J. Chem. Phys.* **118,** 9653–9663 (2003).
8. Stratmann, R. E., Zurales, R. W. & Lucchese, R. R. Multiplet-Specific Multichannel Electron-Correlation Effects in the Photoionization of NO. *J. Chem. Phys.* **104,** 8989–9000 (1996).
9. Wallace, S., Dill, D. & Dehmer, J. L. Shape Resonant Features in the Photoionization Spectra of NO. *J. Chem. Phys.* **76,** 1217–1222 (1982).
10. Huber, K. & Herzberg, G. *Molecular Spectra and Molecular Structure. IV. Constants of Diatomic Molecules* (Springer US, New York, 1979).
11. Dunning, T. H. Gaussian basis Sets for Use in Correlated Molecular Calculations. I. The Atoms Boron Through Neon and Hydrogen. *J. Chem. Phys.* **90,** 1007–1023 (1989).
12. Kendall, R. A., Dunning, T. H. & Harrison, R. J. Electron Affinities of the First-Row Atoms Revisited. Systematic Basis Sets and Wave Functions. *J. Chem. Phys.* **96,** 6796–6806 (1992).
13. Stratmann, R. E. & Lucchese, R. R. A Graphical Unitary Group Approach to Study Multiplet Specific Multichannel Electron Correlation Effects in the Photoionization of O2. *J. Chem. Phys.* **102,** 8493–8505 (1995).
